# Equine Rabies in Southern Colombia, 2024–2025

**DOI:** 10.3390/ani16132020

**Published:** 2026-07-02

**Authors:** Ivan Camilo Sanchez-Rojas, D. Katterine Bonilla-Aldana, Catherin Lorena Solarte-Jimenez, Jorge Luis Bonilla-Aldana, Diana Patricia Dallos-Rodriguez, Lysien I. Zambrano, Alfonso J. Rodriguez-Morales

**Affiliations:** 1 Grupo de Investigación en Genética, Biodiversidad y Manejo de Ecosistemas (GEBIOME), Universidad de Caldas, Manizales 170004, Colombia; ivan.sanchez@itp.edu.co; 2 College of Medicine, Korea University, Seoul 02841, Republic of Korea; dbonilla@korea.ac.kr; 3 Grupo de Investigación en Producción y Salud Animal, Universidad de la Amazonia, Florencia 180001, Colombia; catherin9420@gmail.com; 4 Grupo de Virologia, Universidad El Bosque, Bogotá 111321, Colombia; jlbonilla@unbosque.edu.co; 5 Technical Directorate of Epidemiological Surveillance, Instituto Colombiano Agropecuario (ICA), Bogota 110931, Colombia; diana.dallos@ica.gov.co; 6 Department of Morphological Sciences, School of Medical Sciences, Universidad Nacional Autónoma de Honduras, Tegucigalpa 11101, Honduras; lysien.zambrano@unah.edu.hn; 7 Faculty of Health Sciences, Universidad Científica del Sur, Lima 15074, Peru; 8 Grupo de Investigación Biomedicina, Faculty of Medicine, Fundación Universitaria Autónoma de las Américas-Institución Universitaria Visión de las Américas, Pereira 660003, Colombia

**Keywords:** equine rabies, sylvatic rabies, *Desmodus rotundus*, veterinary surveillance, one health, zoonotic diseases, colombia

## Abstract

Rabies remains a fatal zoonotic disease of major public health and veterinary importance in Latin America. Although canine-mediated rabies has markedly declined in Colombia, sylvatic transmission persists in rural regions, and information on equine rabies in Putumayo is scarce. This study aimed to describe the epidemiological, clinical, gross pathological, and surveillance characteristics of laboratory-confirmed equine rabies cases in Putumayo, southern Colombia, during 2024–2025. A retrospective descriptive study was conducted using national surveillance data. Thirteen equids were identified during outbreak investigations. Five were classified as suspected cases, and all of them died; four were laboratory-confirmed. The remaining animals also died but were not reported to the Colombian Agricultural Institute (ICA) for sample collection; however, they exhibited clinical signs consistent with rabies. Epidemiological and clinical variables, necropsy findings, and diagnostic timelines were analyzed. Laboratory confirmation was performed using direct immunofluorescence and histopathology. The four confirmed cases occurred in four separate outbreaks. All affected equines were unvaccinated and raised under extensive management systems. Progressive neurological deterioration led to 100% case fatality. Pathological findings consistently demonstrated central nervous system involvement. The median times from symptom onset to notification were 7.5 days, and from notification to laboratory diagnosis were 9.0 days. This study provides the first detailed characterization of equine rabies in Putumayo, consistent with sylvatic-origin rabies and highlighting gaps in preventive vaccination. Strengthening integrated surveillance and One Health strategies is essential to reduce the rabies burden.

## Simple Summary

Rabies is a deadly disease that affects both animals and people. Although major progress has been made in controlling rabies spread by dogs in Colombia, transmission from wildlife, especially vampire bats, continues to threaten livestock in rural areas. This study describes four confirmed cases of rabies in horses and mules from Putumayo, southern Colombia, between 2024 and 2025. All affected animals had not been vaccinated against rabies and developed severe neurological signs, including weakness, difficulty walking, paralysis, and inability to stand. Every infected animal died, highlighting the devastating impact of the disease once symptoms appear. Laboratory testing confirmed rabies infection, and investigations suggested that transmission was linked to wildlife. The study also identified delays between the onset of illness and reporting to animal health authorities, emphasizing opportunities to improve surveillance and early detection. These findings provide important information about a disease that remains poorly documented in horses in Colombia. Strengthening vaccination programs, improving disease reporting, and increasing collaboration between animal health, environmental, and public health sectors are essential to prevent future cases, protect livestock, reduce economic losses for rural communities, and lower the risk of exposure to people.

## 1. Introduction

Rabies remains one of the most lethal zoonotic diseases worldwide, causing acute and progressive encephalitis in mammals and leading almost invariably to death once clinical signs develop [1,2,3]. The disease is caused by the rabies virus (RABV), a neurotropic virus belonging to the genus *Lyssavirus* within the family *Rhabdoviridae*. Despite the availability of effective preventive measures, rabies continues to pose a major public health, veterinary, and socioeconomic burden, particularly in low- and middle-income countries [4]. In Latin America, sustained efforts have substantially reduced canine-mediated rabies; however, sylvatic transmission cycles, especially those involving hematophagous bats, remain a persistent and emerging threat to livestock and human populations [2,5,6,7].

In rural and peri-rural settings of South America, the common vampire bat (*Desmodus rotundus*) plays a central role in the maintenance and transmission of RABV [8,9,10,11]. Through repeated blood-feeding behavior, infected bats efficiently transmit the virus to herbivores, including cattle, horses, and other domestic animals. Among these species, equines are particularly vulnerable due to their frequent exposure in open grazing systems and their proximity to forested and fragmented landscapes that favor roosting and foraging by bats. Infected horses usually develop a paralytic or furious form of rabies, progressing rapidly to respiratory failure and death, resulting in substantial economic losses and potential occupational exposure for owners, veterinarians, and animal handlers [12,13].

Over the last two decades, Brazil has produced extensive epidemiological evidence demonstrating the widespread distribution and spatiotemporal clustering of equine rabies, highlighting its relevance as an endemic and neglected disease [3,12,14]. Similar patterns have been reported in other regions of Latin America, Africa, and Asia, emphasizing the global importance of rabies in equids. These studies have underscored the influence of environmental change, deforestation, livestock expansion, and cross-border animal movement on viral circulation and disease emergence. In contrast, information on equine rabies in Colombia remains remarkably scarce [3,15].

In Colombia, rabies continues to circulate in both urban and sylvatic cycles [6,9,16]. While urban outbreaks linked to dogs have been historically documented and progressively controlled, sylvatic rabies transmitted by bats persists as a major concern in rural areas [17,18,19,20,21]. In urban and periurban settings, the emergence of rabies in cats represents a concerning trend [21]. Most published research in the country has focused on bovine rabies, describing its geographical distribution, temporal trends, and associated ecological factors [6]. These studies have demonstrated sustained viral circulation in several departments and highlighted the need for strengthened surveillance and prevention strategies [4]. However, comparable investigations in equine populations are notably lacking, despite the ecological and epidemiological similarities between cattle and horses in endemic regions [22,23,24].

Putumayo, located in the southwestern Colombian Amazon, is a high-risk setting for zoonotic and vector-borne diseases due to its dense rainforests, extensive livestock activities, and intense cross-border mobility with Ecuador and Peru [25,26,27,28]. The region has been historically affected by multiple emerging and re-emerging infections, including yellow fever and sylvatic rabies [28,29]. Nevertheless, systematic documentation of rabies cases in equines from this department has not been previously reported in the scientific literature.

In this context, we describe four fatal cases of laboratory-confirmed equine rabies detected in Putumayo, southern Colombia. By presenting their clinical, epidemiological, and diagnostic characteristics, this study aims to contribute to the limited national and regional evidence on equine rabies, strengthen veterinary surveillance, and support One Health-based strategies for prevention and control in endemic regions.

## 2. Methods

### 2.1. Study Design and Case Definition

**Figure 1:**
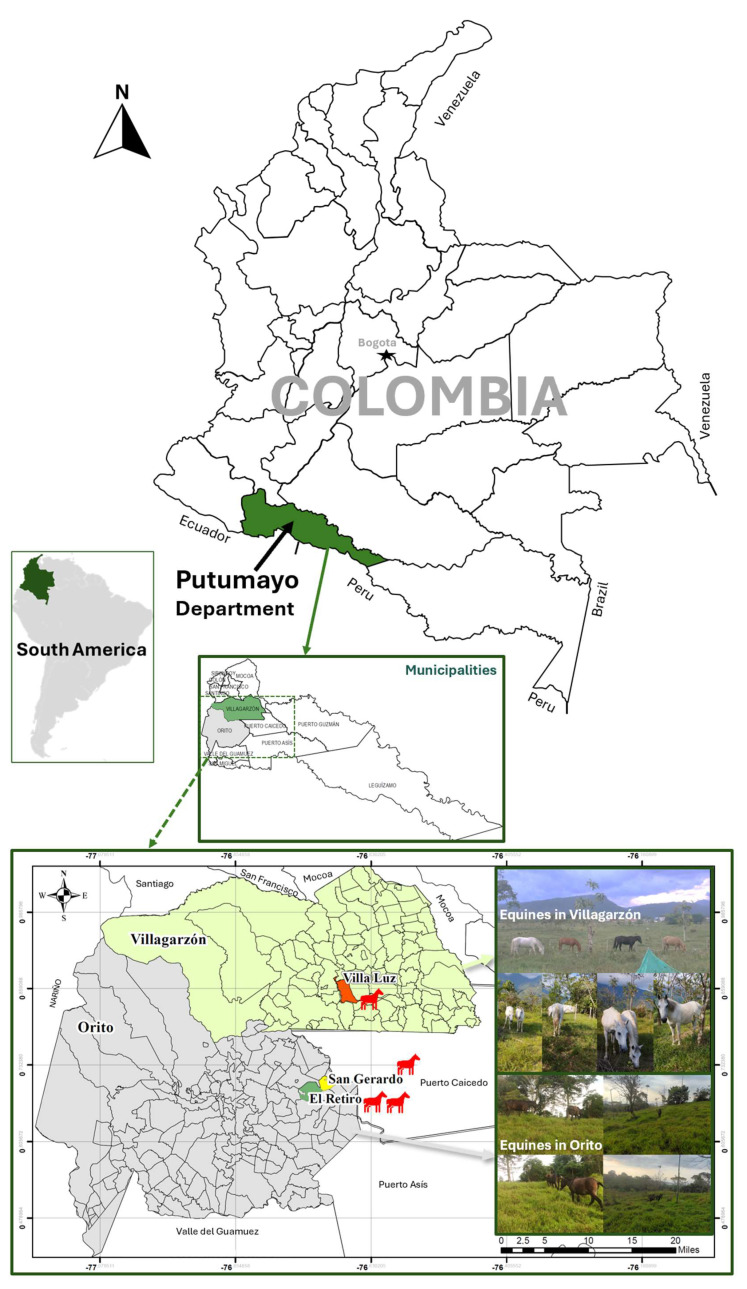
Map of Colombia and its Putumayo department, showing the villages where equine rabies was identified.

A retrospective descriptive study was conducted to characterize suspected and laboratory-confirmed equine rabies cases reported in the Department of Putumayo, southern Colombia, between December 2024 and October 2025. During this period, thirteen equids exposed to the virus were identified in three rural villages (*veredas*) in the municipalities of Orito and Villagarzón: El Retiro, San Gerardo, and Villa Luz (Figure 1). Of these, five were classified as suspected cases, and all had a fatal outcome; four of the deceased animals were laboratory-confirmed for infection with the Rabies virus and included in the present analysis.

A suspected case of equine rabies was defined as any equid presenting with acute neurological signs compatible with rabies, including paralysis, ataxia, incoordination, gait abnormalities, recumbency, progressive weakness, behavioral alterations, or inability to stand, occurring in an area with known circulation of vampire bats (*Desmodus rotundus*), a history of bat bites, or epidemiological linkage to a confirmed rabies outbreak. (https://www.ica.gov.co/areas/pecuaria/servicios/enfermedades-animales/rabia-silvestre-1/epidemiologia-rabia-silvestre.aspx) (accessed on 20 June 2026).

A confirmed case was defined as a suspected case with laboratory confirmation of rabies virus infection by official diagnostic methods performed by the Colombian Agricultural Institute (ICA), including direct immunofluorescence testing of brain tissue.

### 2.2. Data Sources and Variables

Epidemiological, clinical, and laboratory data were obtained from the Colombian Agricultural Institute (ICA) surveillance system, field investigation reports, standardized outbreak notification forms, and structured databases.

The following variables were collected: year of occurrence, municipality and vereda, date of symptom onset, date of notification, outbreak identification, number of exposed animals, number of suspected and confirmed cases, vaccination status, probable origin of infection, and implementation of control measures.

Clinical variables included general condition, locomotor alterations, neurological signs, behavioral changes, disease progression, and outcome. Pathological variables comprised gross and microscopic findings affecting the central nervous system and other organs. For each case, the following surveillance intervals were calculated and analyzed: (i) the time from disease onset to notification of animal health authorities; (ii) the time from notification to field investigation and initial sample collection; (iii) the time from sample collection to laboratory diagnostic results, including direct immunofluorescence, histopathology, and antigenic typing; and (iv) the time from notification to final laboratory diagnosis.

### 2.3. Field Investigation and Necropsy

Following the notification, veterinary officers conducted on-site investigations, including clinical assessments, epidemiological evaluations, and necropsies. Necropsies were performed whenever carcass condition permitted pathological examination, using standardized biosafety procedures. Macroscopic evaluation focused on the brain and meninges, assessing congestion, hyperemia, hemorrhages, edema, and cerebrospinal fluid volume. Peripheral organs were also examined to exclude alternative causes of neurological disease.

### 2.4. Laboratory Diagnosis and Viral Characterization

Brain tissue samples were collected using sterile instruments and standard biosafety procedures from fatal cases and submitted to the authorized reference laboratory (*Laboratorio Nacional de Diagnóstico Veterinario del Instituto Colombiano Agropecuario*). Rabies diagnosis was performed using a direct immunofluorescence assay (DIF) to detect viral antigen, considered the gold standard. All laboratory procedures were performed at the National Veterinary Diagnostic Laboratory of the Colombian Agricultural Institute (ICA) in accordance with standardized national reference protocols for rabies diagnosis and antigenic characterization. Because this study was based on retrospective surveillance records, detailed information on reagent manufacturers, antibody clones, and lot numbers was unavailable for analysis.

Histopathological analyses were conducted on brain tissues to evaluate microscopic lesions compatible with viral encephalitis. In addition, antigenic typing of rabies virus isolates was performed by indirect immunofluorescence (IIF) using infected brain tissue from each case. Only laboratory-confirmed cases were included in the final analysis.

### 2.5. Ethical Considerations

All procedures were conducted in accordance with national veterinary surveillance and public health regulations. No experimental interventions were performed, and data were analyzed anonymously for research purposes.

## 3. Results

### 3.1. Epidemiological and Temporal Characteristics

**Figure 2:**
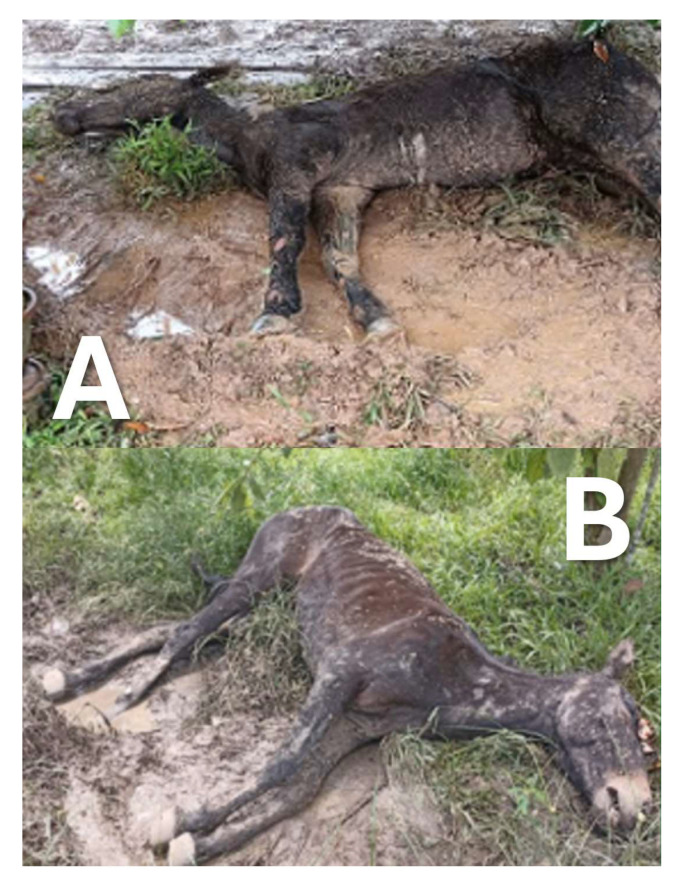
Images of some of the dead equines with laboratory-confirmed rabies in Putumayo. (**A**) from San Gerardo (2025) (*Equus asinus* × *Equus caballus*); (**B**) from Villa Luz (2025) (*Equus asinus* × *Equus caballus*) (Source: ICA Report of Equine Rabies Cases).

Between December 2024 and October 2025, five animals met the suspected case definition for equine rabies in the department of Putumayo, southern Colombia. Four of these cases were laboratory-confirmed, whereas one remained unconfirmed because the carcass was found in an advanced state of decomposition, which precluded the collection of adequate brain tissue samples for laboratory diagnosis. The four laboratory-confirmed cases are described in detail below (Figure 2). One case occurred in late 2024 (a horse, *Equus ferus caballus*), while three cases were reported in 2025 (one horse and two mules, *Equus asinus* × *Equus caballus*). All events were classified by the Colombian Agricultural Institute (ICA) as cases of sylvatic-origin rabies.

The confirmed cases corresponded to four epidemiological outbreaks. Three outbreaks involved a single equid, while one outbreak, reported in 2025 in Villagarzón, affected two animals simultaneously (in one, it was not possible to obtain samples) (Figure 1). The cases were distributed across the municipalities of Orito and Villagarzón (Figure 1), within rural settlements characterized by mixed agricultural–livestock systems and proximity to forested areas (Table 1).

All affected equines were maintained under extensive or semi-extensive management systems and were primarily used for agricultural and transport activities. None of the animals had documented records of prior rabies vaccination (Table 1).

The onset of clinical signs ranged from 25 December 2024 to 11 October 2025. All suspected cases were reported by the owners to the local ICA offices, thereby activating sanitary response protocols, epidemiological investigation (surveillance is carried out jointly with the health sector, within the framework of Decree 780 of 2016), outbreak control, and follow-up measures (Table 1). Field visits and sample collection were generally conducted within 1–3 days after notification, and laboratory confirmation was obtained within days to weeks (critical route of 6–9 days) (Table 2).

Analysis of operational surveillance intervals showed that the median time from the onset of clinical signs to notification by equine owners to local animal health authorities was 7.5 days (interquartile range [IQR]: 3.8–13.3 days). Following notification, field visits and initial sampling were conducted rapidly, with a median time of 1.0 day (IQR: 0.8–1.3 days). The median time from sampling to availability of direct immunofluorescence results was 7.0 days (IQR: 6.8–7.5 days), while histopathological results required a median of 15.0 days (IQR: 11.8–17.3 days). Antigenic typing by indirect immunofluorescence was completed after a median of 11.5 days (IQR: 10.3–17.0 days). Overall, the median interval between notification and final laboratory diagnosis was 9.0 days (IQR: 7.8–10.0 days), reflecting efficient coordination between field surveillance and diagnostic services (Table 2).

### 3.2. Outbreak Characteristics, Morbidity, and Mortality

The number of equids exposed per outbreak ranged from 1 to 9 (Table 1). Morbidity rates varied according to herd size, ranging from 11.1% in the largest outbreak to 100% in outbreaks involving single or paired animals.

All clinically affected equines died, resulting in a case fatality rate of 100% across all outbreaks. Overall mortality and lethality were 100%, reflecting the uniformly fatal outcome of rabies infection once neurological signs became evident. No cases of clinical recovery or prolonged survival were documented (rabies case fatality rate is almost 100% in humans and animals; the equines are highly susceptible to this disease, with rapid neurological progression and a high fatality rate).

### 3.3. Clinical Manifestations

All affected equines developed an acute and progressive neurological syndrome consistent with rabies infection. Initial manifestations included marked prostration, depressed general condition, and reduced responsiveness (Table 3).

As the disease progressed, animals exhibited claudication, altered gait, postural instability, and progressive motor incoordination. These signs rapidly evolved toward severe neurological dysfunction, including complete loss of standing ability and prolonged recumbency.

Involuntary pedal movements in the decubitus position, tremors, muscular fasciculations, and posterior limb paralysis characterized advanced stages. Several animals developed severe weakness and hypersensitivity to external stimuli. Terminal phases were frequently accompanied by cachexia secondary to the inability to feed, reflecting rapid clinical deterioration.

The frequency and distribution of the main clinical manifestations are summarized in Table 3. In all cases, the disease course was acute, and death occurred within a few days after the onset of neurological signs. No animals showed evidence of clinical stabilization or improvement before death.

### 3.4. Gross Pathological Findings

Necropsies were performed in the four laboratory-confirmed cases. Gross pathological evaluation consistently revealed alterations of the central nervous system (Table 4).

The most frequent macroscopic finding was generalized cerebral congestion, accompanied by marked hyperemia of the meninges and cerebral cortex. In addition, a notable increase in cerebrospinal fluid volume was observed during cranial cavity opening in most cases, compatible with acute inflammatory processes affecting the central nervous system.

Severe vascular congestion and petechial hemorrhages were documented in several encephalic structures, and diffuse cerebral edema was observed in some animals. No significant lesions were detected in peripheral organs that could explain the neurological syndrome.

A summary of the main pathological findings is presented in Table 4. These macroscopic lesions were consistent with acute viral encephalitis and supported the diagnosis of rabies in conjunction with laboratory confirmation.

### 3.5. Laboratory Confirmation and Surveillance Response

The reference laboratory (*Laboratorio Nacional de Diagnóstico Veterinario del ICA*) confirmed four cases in accordance with national diagnostic protocols. Brain tissue samples collected during field investigations tested positive for the rabies virus (Table 4). Direct immunofluorescence testing demonstrated the presence of rabies virus antigen in brain tissue samples from all four confirmed cases. However, because the study relied on retrospective surveillance records, detailed descriptions of fluorescence patterns and laboratory imaging documentation were unavailable for analysis.

Laboratory confirmation was achieved using standardized diagnostic techniques, including direct immunofluorescence and antigenic typing by indirect immunofluorescence, in accordance with national surveillance guidelines. Similar diagnostic findings were obtained in all cases, confirming active rabies virus infection and supporting their classification as sylvatic rabies outbreaks (Table 4).

Following laboratory confirmation, comprehensive sanitary and epidemiological measures were implemented in all affected premises. These included activating territorial zoonosis councils to coordinate surveillance activities across the health and environment sectors, and capturing hematophagous bats on properties with fresh bites in areas where outbreak-control activities are underway.

Epidemiological investigations were conducted to identify potential sources of exposure and assess the extent of viral circulation. Owners, animal handlers, and other exposed individuals were referred to public health authorities for risk assessment and, when indicated, post-exposure prophylaxis.

## 4. Discussion

This study documents four laboratory-confirmed cases of equine rabies in the department of Putumayo, southern Colombia, between 2024 and 2025. Although the clinical and pathological manifestations observed are consistent with previous reports from other endemic regions, published information on equine rabies from Putumayo and the Colombian Amazon remains extremely limited. Therefore, this case series contributes novel epidemiological, surveillance, and field-based evidence from an underreported region where the disease has been poorly documented. Although rabies remains endemic in Colombia, published information on its occurrence in horses is extremely limited [6,9,11,16,20]. Therefore, the present case series provides novel evidence on the circulation of rabies virus in equine populations within a highly biodiverse and ecologically dynamic area of the Colombian Amazon, consistent with the probable involvement of sylvatic transmission cycles in rural settings. However, direct molecular evidence linking cases to specific wildlife reservoirs was unavailable.

The temporal distribution of cases, with one event in 2024 followed by three in 2025, may be compatible with continued viral circulation in local wildlife reservoirs, although the limited number of cases and absence of molecular data preclude definitive conclusions regarding transmission dynamics [11]. This pattern may be consistent with enzootic maintenance of rabies virus in wildlife reservoirs, particularly hematophagous bats, which are widely distributed in the region [6,8,11,14,16,21]. Putumayo’s extensive forest cover, fragmented landscapes, and expanding agricultural frontier create favorable conditions for bats to roost, forage, and interact with domestic animals. These ecological characteristics, combined with extensive livestock management systems, likely increase the vulnerability of equine populations to repeated exposure [6,30].

Previous national-scale modeling analyses based on municipality-level rabies outbreaks from 1982 to 2010 demonstrated that sylvatic rabies transmission in Colombia is geographically heterogeneous, with clusters of high incidence in some regions and few reported events in large areas, including parts of the Amazon [29]. These studies highlighted that low notification rates in remote departments may reflect limitations in surveillance and diagnostic capacity rather than the true absence of viral circulation [29]. Using environmental variables and ecological niche modeling, temperature and precipitation patterns, particularly during dry seasons, were identified as key drivers of the suitability for *Desmodus rotundus* infection [10,11,29]. Although the highest-incidence municipalities were located mainly outside Putumayo, the models supported the plausibility of sylvatic rabies risk in Amazonian settings where ecological conditions are favorable, but historical reporting has been limited [29]. In this context, the present case series complements these predictive assessments.

Interpretation of the number of laboratory-confirmed cases should consider the geographic and operational characteristics of Putumayo [27,28,31]. Large rural extensions, difficult access to farms, dependence on river and secondary-road transportation, and proximity to international borders may limit timely notification and diagnostic investigation of suspected rabies cases. Consequently, the number of laboratory-confirmed cases likely represents only a fraction of the total burden of equine rabies occurring in the region. Similar surveillance challenges have been recognized in remote areas of Latin America, where logistical barriers may reduce sample submission rates and laboratory confirmation of animal rabies cases.

The geographic clustering of cases in Orito and Villagarzón (Figure 1) further supports the role of local ecological and environmental factors in shaping rabies transmission dynamics [9,11]. Mixed agricultural activities, proximity to riparian forests, and limited physical barriers between wildlife habitats and grazing areas characterize both municipalities [31,32]. Similar spatial patterns have been described in other endemic regions, where landscape configuration, deforestation, and land-use change facilitate contact between the reservoir species and domestic hosts. In this context, equines may function as sentinel species, reflecting underlying viral circulation in surrounding ecosystems [12,33].

All affected animals in this series were unvaccinated, highlighting a persistent gap in preventive practices among equine owners in rural Colombia. Although rabies vaccination is widely promoted for dogs and cattle, horses are frequently overlooked in routine immunization programs across species, with administration handled by the producer (Resolution 9028 of 2024). [4,34]. This omission may be related to limited awareness of disease risk and economic constraints. There are geographical and access barriers that may affect vaccination. The absence of vaccination in all cases underscores the preventable nature of these fatal events and emphasizes the need to strengthen immunization coverage in at-risk areas [35]. Even before 2025, the availability of equine vaccines was limited, which restricted immunization strategies. In 2025, a new vaccine for this species was registered with the ICA (Colombian Agricultural Institute) and is now commercially available to producers.

In the department of Putumayo, the Instituto Colombiano Agropecuario (ICA) has implemented vaccination activities on rural farms against wildlife-associated rabies in susceptible species, particularly cattle and buffalo, with records available since 2017. According to these data, 176,172 animals were vaccinated during the first cycle of 2017, and 928 were vaccinated during the second cycle of the same year. Subsequently, vaccination coverage varied, ranging from 14,645 animals (first cycle of 2018) to 46,917 (first cycle of 2023) per cycle.

During the period associated with the equine rabies outbreaks reported here, 1813 animals were vaccinated in the second cycle of 2024 and 45,008 in the first cycle of 2025. These records reflect the sustained implementation of immunization activities as the primary preventive and control measure in the region. However, the confirmation of rabies cases in equids highlights the persistence of epidemiological risk and the need to strengthen epidemiological surveillance, implement targeted vaccination in susceptible species, and enhance risk communication in endemic areas.

The uniformly fatal outcome observed in this series is consistent with the natural history of rabies once neurological signs appear [1,2,4,21]. All affected equines developed rapidly progressive disease, culminating in death within a short period. The high case fatality rate reflects the intrinsic virulence of the rabies virus, and there are no effective therapeutic options at advanced stages globally for animals or humans. These findings reinforce the critical importance of prevention and early detection [36,37].

The clinical presentation documented in this study aligns with classical descriptions of rabies in herbivores. Initial nonspecific signs such as prostration and reduced responsiveness were followed by progressive locomotor impairment and overt neurological dysfunction. Advanced manifestations, including pedal movements, posterior limb paralysis, and prolonged recumbency, reflected severe central nervous system involvement. The presence of cachexia in terminal stages further illustrates the rapid deterioration associated with feeding incapacity and systemic exhaustion. Although aggressive behavior is often emphasized in rabies descriptions, particularly in carnivores, the predominantly paralytic form observed here is typical of infection in equines and other herbivores [38,39,40].

Postmortem findings provided additional support for the diagnosis and pathophysiological mechanisms of disease. Generalized cerebral congestion, meningeal hyperemia, increased cerebrospinal fluid volume, and petechial hemorrhages were consistently observed, indicating acute inflammatory and vascular disturbances within the central nervous system. These lesions are compatible with viral encephalitis and reflect disruption of the blood–brain barrier, neuronal injury, and immune-mediated responses. The absence of significant lesions in peripheral organs further supports the neurotropic nature of the rabies virus and its primary localization within neural tissues [41,42].

Laboratory confirmation using standardized diagnostic methods ensured the reliability of case classification. Direct immunofluorescence assays remain the gold standard for rabies diagnosis in animals and offer high sensitivity and specificity. The consistency of laboratory findings across all cases supports the homogeneity of infection and confirms active viral replication. The integration of field investigation, necropsy, and laboratory analysis illustrates the effectiveness of the surveillance framework when timely reporting occurs [43,44].

A notable strength of this series is the prompt notification by animal owners and the rapid response by veterinary authorities. Early reporting facilitated immediate field investigation, sample collection, and implementation of control measures. The responsiveness observed in these cases reflects the impact of ongoing surveillance efforts and highlights the importance of community engagement in disease control. This was further supported by analysis of operational time intervals, which showed a median delay of 7.5 days between symptom onset and notification, followed by a rapid field response within a median time of 1 day. These findings indicate a rapid initial recognition and strong institutional capacity for prompt follow-up once cases are reported [34,45,46].

Despite this effective response, the occurrence of multiple cases over consecutive years indicates that surveillance alone is insufficient to interrupt transmission. Persistent circulation in wildlife reservoirs requires integrated approaches addressing ecological, veterinary, and public health dimensions. In regions such as Putumayo, where human activities increasingly encroach on natural habitats, traditional compartmentalized control strategies are unlikely to achieve sustained success. Instead, coordinated interventions are needed to address bat ecology, livestock management, vaccination coverage, and community education [4,8,17,35].

The classification of all cases as sylvatic-origin rabies is consistent with the recognized importance of bat-associated transmission in Colombia [17,47]. While canine rabies has been substantially reduced through vaccination campaigns, sylvatic cycles continue to represent a public health challenge [48]. Hematophagous bats play a significant ecological role, as they are integral to trophic dynamics and contribute to the natural balance of wildlife populations. In addition, they exhibit high mobility, complex social structures, and the capacity to exploit anthropogenic landscapes, which makes eradication technically and ecologically infeasible, since not all bat groups are infected with the Rabies virus, as viral circulation depends on specific epidemiological and environmental conditions.

At the same time, ecological and ecosystem-level factors are also important. The concept of One Health includes environmental health as a key component of this balance, including in the context of rabies. Consequently, control strategies should focus on epidemiological surveillance, timely vaccination of susceptible species, and technically justified selective vector control, prioritizing the reduction of transmission risk rather than the indiscriminate elimination of reservoir populations [9,10,11].

The findings of this study also have important public health implications. Equines often live in proximity to humans, and infected animals may expose owners, caretakers, veterinarians, and community members through bites, contact with saliva, or handling of carcasses [49,50]. The relatively short median interval between notification and final diagnosis observed in this study reflects efficient coordination between animal health laboratories and field services, which is essential for timely risk assessment and implementation of post-exposure prophylaxis in exposed individuals. The integration of veterinary and medical surveillance systems is therefore essential to ensure timely risk assessment and post-exposure prophylaxis [51].

From a One Health perspective, this case series illustrates the interconnectedness of environmental change, animal health, and human vulnerability. Deforestation, agricultural expansion, and settlement growth alter wildlife behavior and increase interspecific interactions. In Putumayo, these processes are occurring rapidly, driven by economic development and demographic pressures. The emergence of equine rabies in this context reflects broader ecological disruption and serves as an indicator of systemic imbalance. The documented diagnostic timelines further highlight how human, animal, and laboratory health systems interact operationally, reinforcing the importance of integrated surveillance platforms within a One Health framework [52,53].

The limited number of cases represents an inherent constraint of this study. As a descriptive series, it cannot establish causal relationships or quantify risk factors with statistical precision. Although necropsy reports were available and contributed to the pathological characterization of the cases, photographic documentation of gross pathological findings and detailed microscopic descriptions from histopathological examinations were not consistently available because the study was based on retrospective surveillance records. Consequently, representative images of lesions could not be included in the manuscript; pathological characterization was largely restricted to gross necropsy findings, and comparison of microscopic lesions across cases was not possible. Additionally, information on bat population dynamics, roost distribution, and viral variants was unavailable, precluding a more refined ecological interpretation.

Furthermore, molecular characterization and sequencing of rabies virus isolates were not available, preventing phylogenetic analyses that could have clarified the genetic relationships among cases, confirmed transmission linkages, and provided greater insight into the wildlife reservoirs and transmission pathways involved in these outbreaks. Therefore, the attribution of viral maintenance to specific wildlife populations should be interpreted with caution and based primarily on epidemiological and ecological evidence rather than direct molecular confirmation.

Future research should integrate molecular epidemiology, spatial analysis, and ecological modeling to better understand rabies transmission in southern Colombia. Sequencing of viral isolates combined with phylogenetic and phylogeographic analyses could clarify transmission pathways, identify circulating viral lineages, characterize evolutionary relationships among outbreaks, and improve understanding of rabies virus dispersion across the Colombian Amazon and neighboring border regions. Additionally, because laboratory data were obtained from routine surveillance records, detailed information on diagnostic reagents, antibody clones, and manufacturer-specific assay characteristics was unavailable for inclusion in this study. Longitudinal surveillance of bat populations and livestock herds would facilitate early detection of viral circulation and evaluation of intervention effectiveness. Furthermore, socioeconomic studies exploring barriers to vaccination and reporting could inform tailored outreach strategies. Routine monitoring of surveillance performance indicators, such as notification delays and diagnostic turnaround times, should also be incorporated into national rabies control programs to identify operational gaps and guide quality improvement [54,55].

Preventing equine rabies in endemic regions requires sustained political commitment, resource allocation, and intersectoral collaboration. Vaccination programs must explicitly include horses and other equids, particularly in high-risk areas. Training veterinary personnel, expanding diagnostic capacity, and strengthening reporting systems are also critical components. Community participation, supported by culturally appropriate education initiatives, remains fundamental to long-term success. Improving early case recognition by owners and local practitioners may further reduce notification delays and enhance overall system performance [46,56].

### Limitations

This study has several limitations that should be considered when interpreting its findings. First, the small number of laboratory-confirmed cases reflects the descriptive nature of this case series. It limits the ability to perform inferential statistical analyses or identify robust risk factors associated with equine rabies transmission in the region. Although the cases provide valuable baseline information, they may not fully represent the true burden of disease in Putumayo.

Second, the retrospective design relied on routinely collected surveillance data and field reports, which may be subject to incomplete documentation, recall bias, and variability in data quality. Some clinical and epidemiological variables could not be consistently verified for all suspected cases, and information on management practices and environmental exposures was limited. Additionally, the study was conducted in a remote Amazonian border region where geographical barriers, dispersed livestock populations, and transportation limitations may affect disease notification and sample submission. Consequently, some suspected rabies cases may not have undergone laboratory testing, potentially leading to an underestimation of the true burden of equine rabies in Putumayo.

Third, virological characterization was limited to antigenic typing performed as part of the routine national veterinary surveillance protocol, and viral genome sequencing was unavailable for the outbreaks investigated. Consequently, phylogenetic relationships, viral evolutionary patterns, and transmission pathways could not be assessed. Molecular characterization of rabies virus isolates through whole-genome or partial-gene sequencing would provide valuable information regarding viral diversity, circulation of specific lineages, and potential links between outbreaks. Future studies incorporating genomic surveillance would substantially improve understanding of rabies ecology and transmission dynamics in the Colombian Amazon.

Fourth, histopathological evaluation was limited to selected tissues and lacked standardized quantitative scoring. This may have restricted the detailed characterization of microscopic lesions associated with rabies infection.

Finally, moderate delays between symptom onset and notification may have resulted in underdetection of milder or atypical cases. Therefore, additional undiagnosed cases may have occurred in the study area. Despite these limitations, this study provides important initial evidence on equine rabies in southern Colombia and establishes a foundation for future surveillance and research efforts.

## 5. Conclusions

This study provides a detailed characterization of laboratory-confirmed equine rabies cases reported from Putumayo, Colombia, documenting fatal infections classified as sylvatic-origin rabies, uniformly fatal clinical outcomes, and consistent neurological and gross pathological findings in affected animals. By integrating clinical, epidemiological, necropsy-based pathological, and operational surveillance data, this work contributes to the limited evidence available on equine rabies in an ecologically complex region of the Colombian Amazon.

The findings complement previous national modeling evidence by providing contemporary field-based evidence of equine rabies occurrence in Amazonian municipalities where historical reporting has been limited. Although the epidemiological investigations and antigenic characterization were compatible with sylvatic-origin transmission, additional molecular, ecological, and wildlife surveillance studies are needed to characterize transmission pathways and reservoir-host dynamics better. Together, these findings highlight the vulnerability of unvaccinated equine populations in rural settings and reveal persistent gaps in preventive practices. The lack of vaccination among all affected animals underscores the preventable nature of these fatal events. It reinforces the need to strengthen surveillance, risk communication, and vaccination strategies for equids in rabies-transmission areas.

## Figures and Tables

**Table 1:** Epidemiological and Temporal Characteristics of Fatal Suspected and Laboratory-Confirmed Equine Rabies Cases in Putumayo, Colombia (2024–2025).

						Cases				
#	Year	Municipality	Locality	Outbreak ID	Exposed Equids	Suspected	Confirmed	Species	Vaccination Status	Probable Origin
1	2024	Orito	El Retiro	O-01-2024	1	1	1	*Equus ferus caballus*	Not vaccinated	Sylvatic
2	2025	Orito	San Gerardo	O-01-2025	9	1	1	*Equus asinus* × *Equus caballus*	Not vaccinated	Sylvatic
3	2025	Orito	El Retiro	O-02-2025	1	1	1	*Equus ferus caballus*	Not vaccinated	Sylvatic
4	2025	Villagarzón	Villa Luz	V-01-2025	1	1	1	*Equus asinus* × *Equus caballus*	Not vaccinated	Sylvatic
5	2025	Villagarzón	Villa Luz	V-01-2025	1	1	0	*Equus asinus* × *Equus caballus*	Not vaccinated	Sylvatic

**Table 2:** Operational Surveillance and Diagnostic Time Intervals in Equine Rabies Cases in Putumayo, Colombia (2024–2025).

Indicator	Median (Days)	IQR (Days)
Time between disease onset and notification	7.5	3.8–13.3
Time between notification and field visit/first sampling	1	0.8–1.3
Time between sampling and DIF results	7	6.8–7.5
Time between sampling and histopathology results	15	11.8–17.3
Time between sampling and IIF antigenic typing results	11.5	10.3–17.0
Time between notification and final laboratory diagnosis	9	7.8–10.0

IQR, interquartile range. DIF, direct immunofluorescence. IIF, indirect immunofluorescence.

**Table 3:** Main Clinical Manifestations Observed in Equines Suspected and Laboratory-Confirmed for Rabies in Putumayo, Colombia.

Clinical Category	Manifestation	N	%
General condition	Prostration/marked depression	5	100
Behavioral changes	Reduced responsiveness	5	100
Locomotor alterations	Claudication/gait disturbance	5	100
Neurological signs	Ataxia/incoordination	5	100
	Loss of standing ability	5	100
	Posterior limb paralysis	4	80
	Pedaling movements in recumbency	4	80
Neuromuscular signs	Tremors/fasciculations	3	60
Systemic deterioration	Cachexia/inability to feed	3	60
Outcome	Death	5	100

**Table 4:** Gross Pathological and Laboratory Findings in Equines with Laboratory-Confirmed Rabies in Putumayo, Colombia.

Finding Category	Observation	N	%
Central nervous system	Generalized cerebral congestion	4	100
	Meningeal and cortical hyperemia	4	100
	Increased cerebrospinal fluid volume	3	75
	Petechial hemorrhages	3	75
	Diffuse cerebral edema	2	50
Peripheral organs	Significant lesions	0	0
Diagnostic methods	Direct immunofluorescence positive	4	100
	Indirect immunofluorescence positive-Antigenic variant 3	1	25
	Indirect immunofluorescence positive-Antigenic variant 5	3	75
Final diagnosis	Rabies virus infection (RABV)	4	100

## Data Availability

The data presented in this study are available upon request from the corresponding author.
